# Supplementary material for: The Impact of Producing Type and Dietary Crude Protein on Animal Performances and Microbiota Together with Greenhouse Gases Emissions in Growing Pigs
Source: Animals (Basel). 2020 Sep 25;10(10):1742. doi: 10.3390/ani10101742 (PMC7601936; doi:10.3390/ani10101742)
Supplement: Supplementary file 1 [file animals-10-01742-s001.pdf]

animals

Supplementary Materials: The Impact of Producing Type and Dietary Crude Protein on Animal Performances and Microbiota Together with Greenhouse Gases Emissions in Growing Pigs

Ahmad Reza Seradj, Joaquim Balcells *, Laura Sarri, Lorenzo José Fraile and Gabriel de la Fuente Oliver

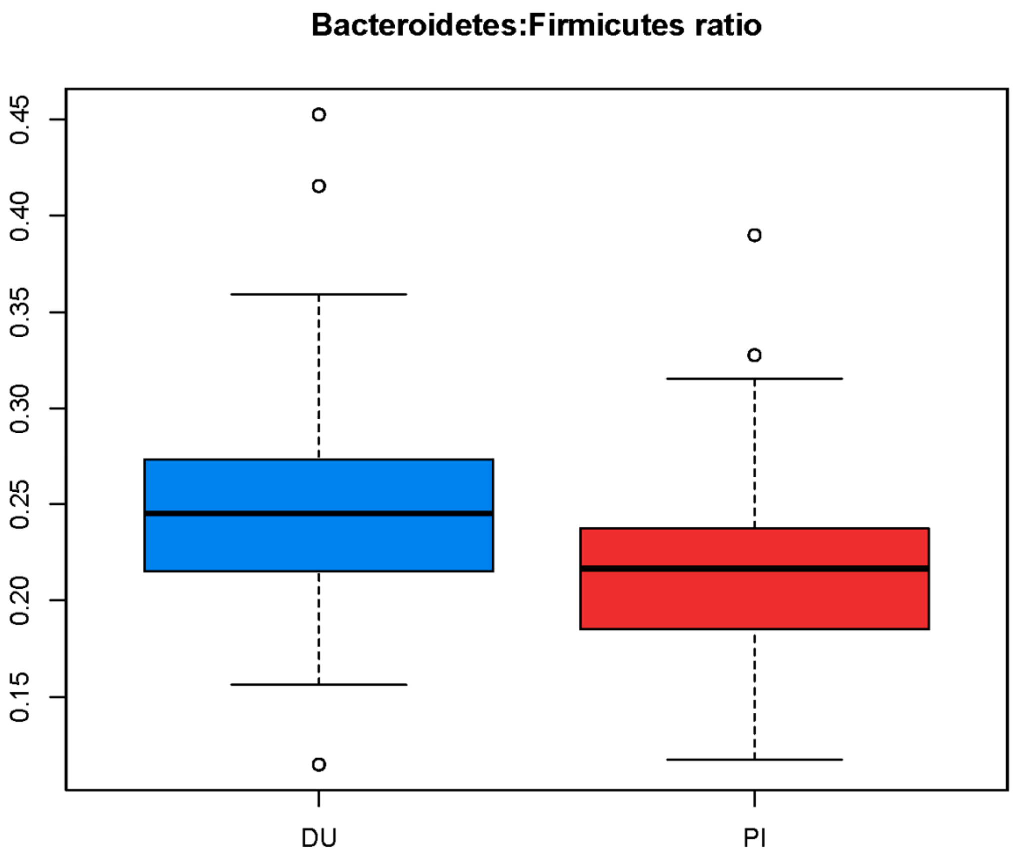

Figure 1. Bacteroidetes to firmicutes ratio in animals belonged to either Duroc (DU) or F2 (PI) producing type.

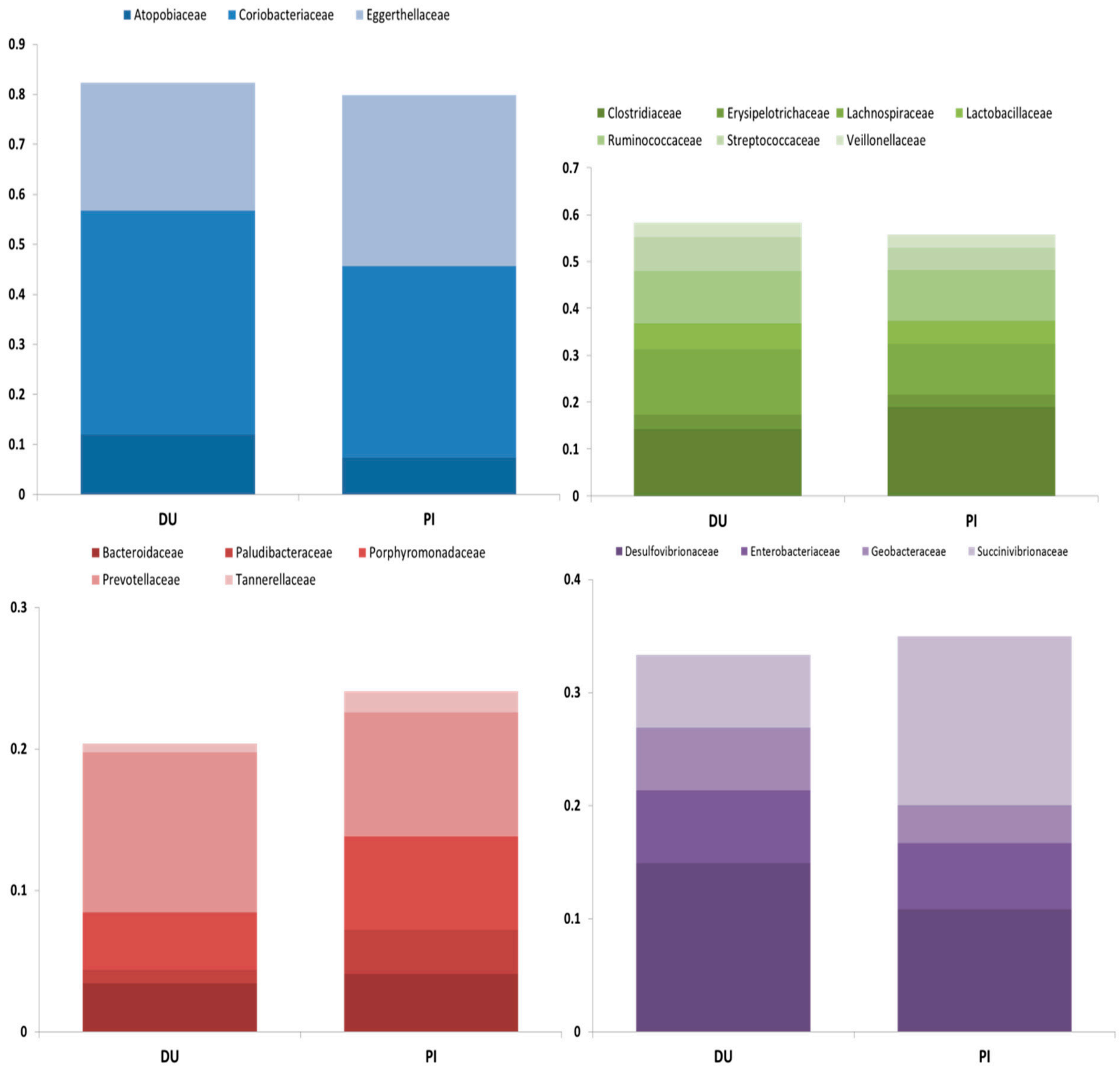

Figure 2. Distribution of families within the main phyla (*Actinobacteria* in blue, *Firmicutes* in green, *Bacteroidetes* in red and *Proteobacteria* in violet) presented in the fecal microbiota of the animals under the study, according to the producing type.

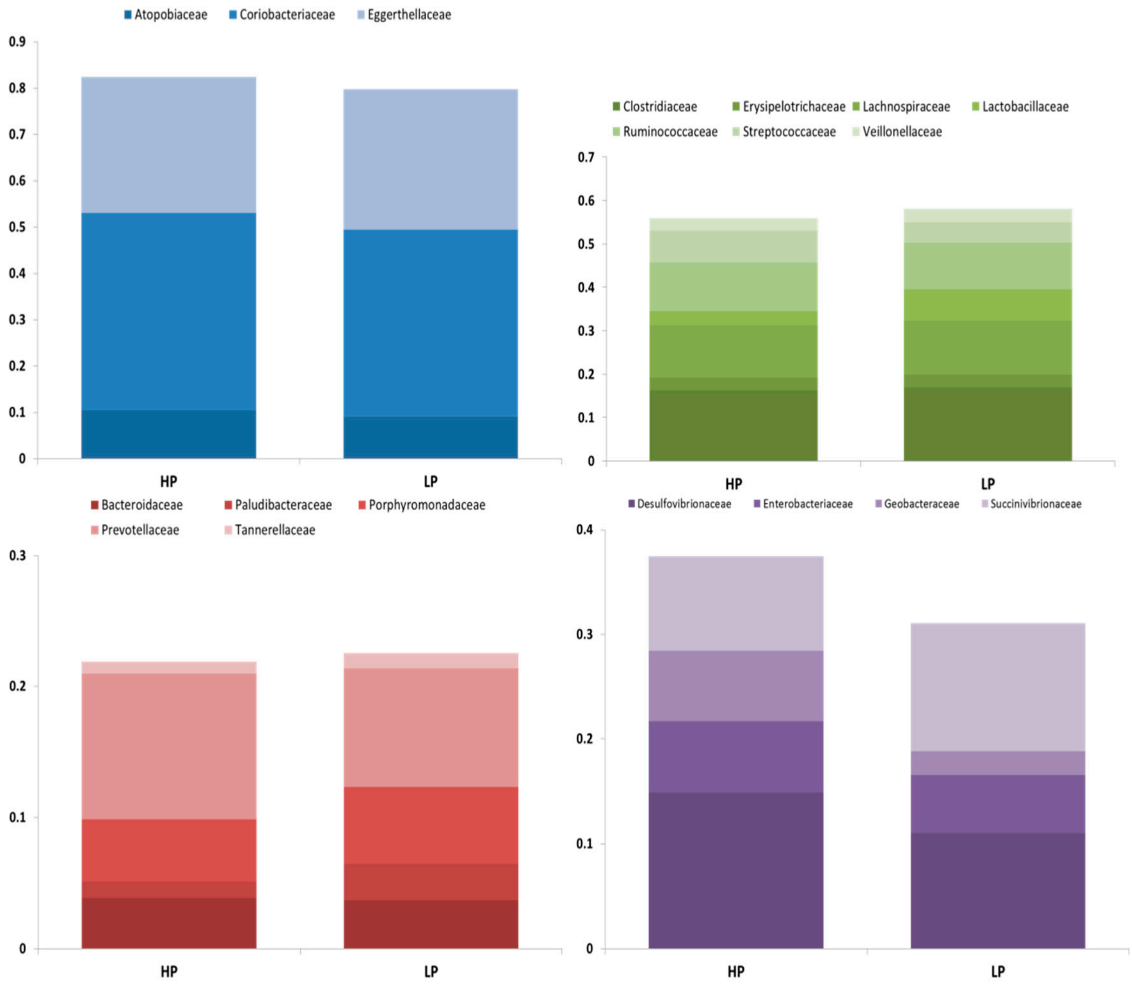

Figure 3. Distribution of families within the main phyla (*Actinobacteria* in blue, *Firmicutes* in green, *Bacteroidetes* in red, and *Proteobacteria* in violet) presented in the fecal microbiota of the animals under the study, according to the level of protein in the diet.

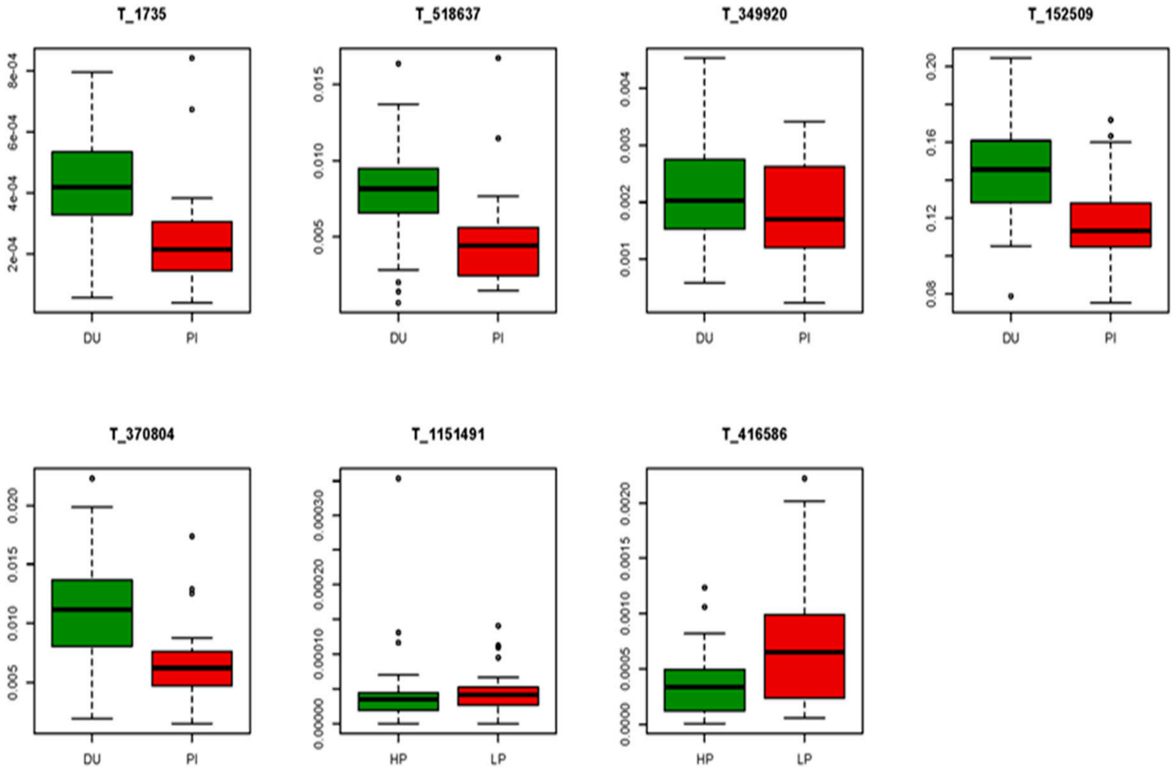

Figure 4. Main discriminative taxons found in the sPLS-DA, with abundance higher than 0.1% (T_1735 = *[Eubacterium] biforme*; T_518637 = *Eubacterium biforme* DSM 3989; T_349920 = uncultured Coriobacteriales bacterium; T_152509= uncultured Bacteroidetes bacterium; T_370804= uncultured Prevotellaceae bacterium; T_1151491= *Blautia sp.* canine oral taxon 143; T_416586= *Selenomonas bovis*).
